# Prediction of lymph node metastasis in early colorectal cancer based on histologic images by artificial intelligence

**DOI:** 10.1038/s41598-022-07038-1

**Published:** 2022-02-22

**Authors:** Manabu Takamatsu, Noriko Yamamoto, Hiroshi Kawachi, Kaoru Nakano, Shoichi Saito, Yosuke Fukunaga, Kengo Takeuchi

**Affiliations:** 1 Division of Pathology, Cancer Institute, Japanese Foundation for Cancer Research, 3-8-31, Ariake, Ko-to-ku, Tokyo, 135-8550 Japan; 2 Department of Pathology, Cancer Institute Hospital, Japanese Foundation for Cancer Research, Tokyo, Japan; 3 Department of Endoscopy, Cancer Institute Hospital, Japanese Foundation for Cancer Research, Tokyo, Japan; 4 Department of Colorectal Surgery, Cancer Institute Hospital, Japanese Foundation for Cancer Research, Tokyo, Japan; 5 Pathology Project for Molecular Targets, Cancer Institute, Japanese Foundation for Cancer Research, Tokyo, Japan

**Keywords:** Colorectal cancer, Machine learning

## Abstract

Risk evaluation of lymph node metastasis (LNM) for endoscopically resected submucosal invasive (T1) colorectal cancers (CRC) is critical for determining therapeutic strategies, but interobserver variability for histologic evaluation remains a major problem. To address this issue, we developed a machine-learning model for predicting LNM of T1 CRC without histologic assessment. A total of 783 consecutive T1 CRC cases were randomly split into 548 training and 235 validation cases. First, we trained convolutional neural networks (CNN) to extract cancer tile images from whole-slide images, then re-labeled these cancer tiles with LNM status for re-training. Statistical parameters of the tile images based on the probability of primary endpoints were assembled to predict LNM in cases with a random forest algorithm, and defined its predictive value as random forest score. We evaluated the performance of case-based prediction models for both training and validation datasets with area under the receiver operating characteristic curves (AUC). The accuracy for classifying cancer tiles was 0.980. Among cancer tiles, the accuracy for classifying tiles that were LNM-positive or LNM-negative was 0.740. The AUCs of the prediction models in the training and validation sets were 0.971 and 0.760, respectively. CNN judged the LNM probability by considering histologic tumor grade.

## Introduction

Endoscopic resection for submucosal invasive (T1) colorectal cancer (CRC) has rapidly increased over the last decade^1,2^. Approximately 10% of T1 CRC can metastasize to lymph nodes, making histologic evaluation for T1 CRC critical for determining the indications for additional surgery^3–5^. Previous studies described five independent histologic risk factors for lymph node metastasis (LNM), as follows; depth of submucosal invasion (≥ 1000 µm), poorly differentiated or mucinous carcinoma, lymphatic invasion, venous invasion, and tumor budding^3–5^. The 2020 Japanese Society for Cancer of the Colon and Rectum guidelines recommend surgical intervention for T1 CRC patients after endoscopic resection when at least one histologic risk factor is present^6^. While this approach minimizes the risk for relapse after endoscopic resection, it may also result in overtreatment because many T1 CRC patients without LNM undergo additional surgery. Indeed, overall relapse rate after endoscopic resection was reported to be 2.3–7.3%^7^, while the incidence of LNM for additional surgery was reported to be 9.7–11.9%^8–10^. Many studies have reported the utility of various histologic risk factors for predicting LNM^11–13^, but the interobserver disagreement among pathologists cannot be completely resolved^14^. To address these issues, a more reproducible and precise prediction system must be developed.

The application of machine-learning techniques including neural networks has expanded in the field of medicine^15–18^. Several machine-learning models for histologic images have been proposed for histologic diagnosis or predicting prognosis^19–25^. Most of these models utilize convolutional neural networks (CNN) for classifying histologic images because of their strong and stable tissue classification ability. One concern, however, is the lack of clarity regarding the relationship between histologic features and the decision process of CNN. When predicting prognosis for cancer patients, it is important for both clinicians and patients to understand why the machine made such a decision. A previous study revealed the relationship between histologic images and prognostic risk stratification of renal cancer by CNN^26^, but no similar studies have been performed for CRC concerning the relationship between histologic patterns and the predictive values of artificial intelligence.

Here we introduce a machine-learning algorithm for predicting LNM for T1 CRC with H&E-stained histologic whole-slide images (WSI), and clarify the decision processes for the predictive values by combining CNN and random forests (RF).

## Results

### Neural network machine-learning models and characteristic tile images

The accuracy and loss of image classifier #1 for classifying cancer and others (2 classes) were 0.980 and 0.039, respectively (Supplementary Fig. 1). When classifying all histologic-type classes, the accuracy and loss were 0.920 and 0.247, respectively. The accuracy and loss of image classifier #2 were 0.740 and 0.524, respectively. (Supplementary Fig. 2).

**Figure 1:**
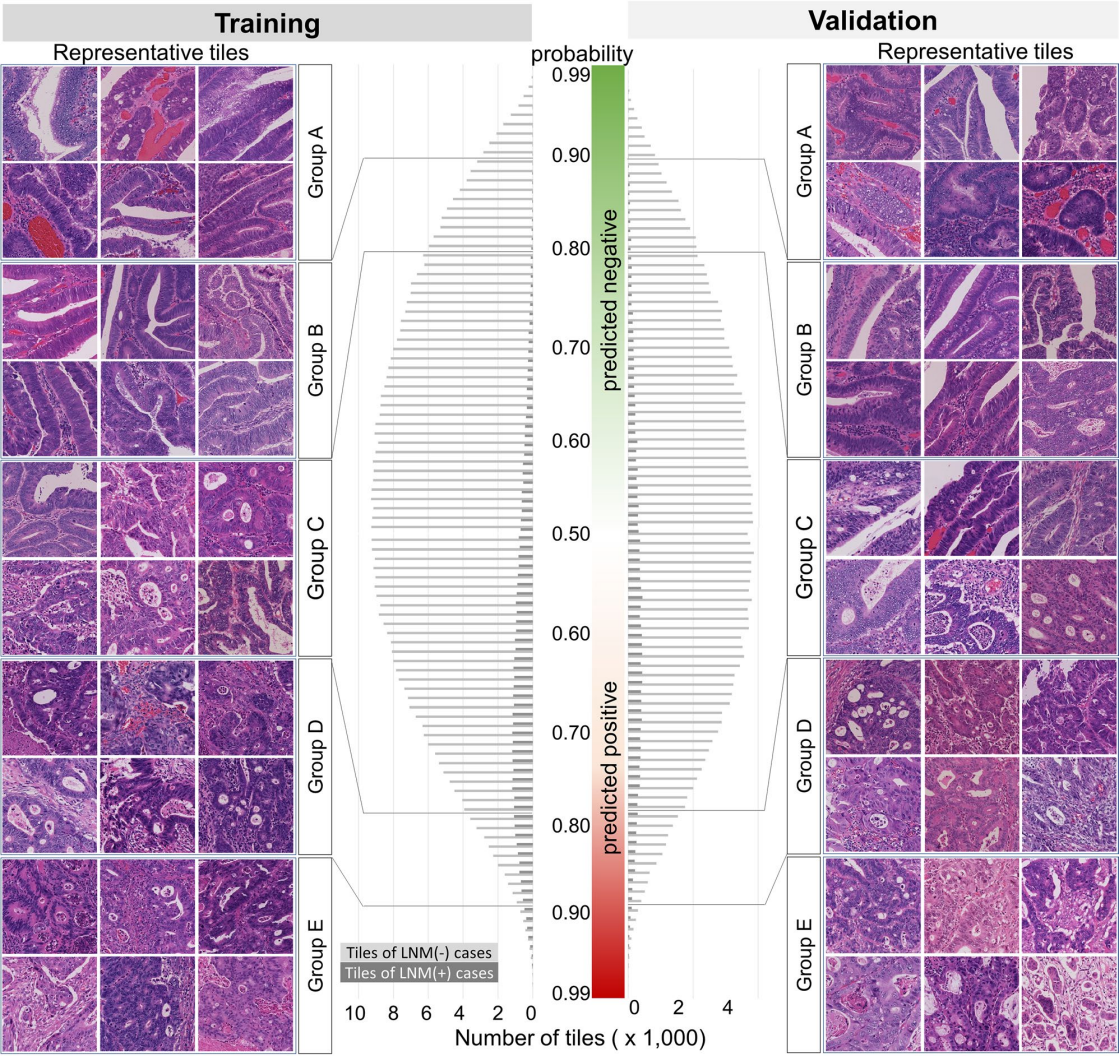
The profiles of tile images in the training and validation datasets. Bars indicate the number of tiles for each probability rank. Representative tile images of the training set are shown on the left side, and those of the validation set are shown on the right side. Note the differences of histologic grade among the groups, which the tile images in Group A show well differentiated tubular adenocarcinoma, while those in Group E contain poorly differentiated cancer cell clusters with desmoplastic stroma. LNM, lymph node metastasis.

The number of tiles sorted by predictive probability for classifier #2 showed a normal distribution (Fig. 1), and the most frequent tile probabilities for the training and validation sets were 0.53 and 0.52 in LNM-negative cases, and 0.73 and 0.57 in LNM-positive cases, respectively (Fig. 1). Histologic characteristics of representative Group A tiles showed large cancer glands with little fibrotic stroma, and Group B tiles resembled those of Group A with more obvious structural atypia. In contrast, Group D tiles showed fused or cribriform cancer glands, and Group E tiles showed severe structural atypia with some isolated cancer cells embedded in desmoplastic stroma, resulting in an architectural complexity. In addition, the grade of nuclear atypia tended to be increased in Groups D and E compared with that in Groups A and B. Group C tiles, unconfidently judged by the classifier, showed a variety of histologic patterns. Importantly, both the training and validation datasets showed similar histologic trends, suggesting that classifier #2 returned the predictive value on the basis of an understandable histologic theory.

### Predictive power of the random forest machine-learning model

**Figure 2:**
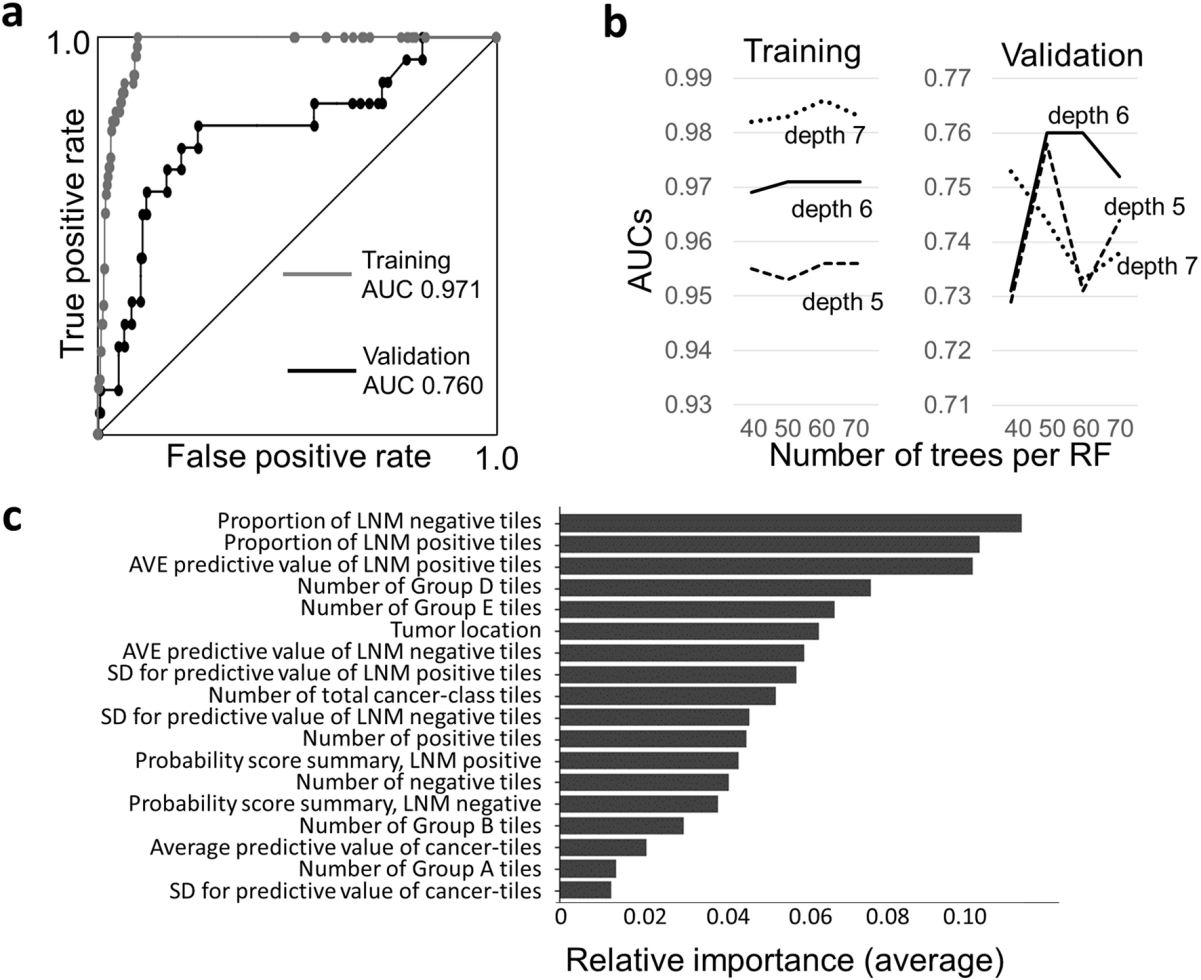
Predictive accuracy of the random forest model. (**a**) Receiver operating characteristics (ROC) curves of the training and validation sets. (**b**) Area under the ROC curves (AUCs) for several conditions. The depth of decision trees for earning a maximum AUC was 7, while that of test set was 6, indicating overfitting of the training set with a depth of 7. RF, random forest. (**c**) Relative importance of 18 parameters. The importance was averaged for 20 random forests. LNM, lymph node metastasis; AVE, average; SD, standard deviation.

The area under the receiver operating characteristics curve (AUC) for predicting LNM in the training and validation sets were 0.971 and 0.760, respectively (Fig. 2a). The specificity and sensitivity were balanced when the RF score was 0.70, and the accuracy of the model for the training and validation sets was 0.91 and 0.75, respectively. The AUCs were highest when the depth and number of RF trees were 6 and 60, respectively (Fig. 2b). The important parameters for predicting LNM were the proportion of tiles predicted as LNM-negative or LNM-positive, average predictive probability of tiles, and number of Group D and E tiles (Fig. 2c). Tumor location was also important for predicting LNM. The differences in these parameters between LNM-negative and LNM-positive cases were statistically significant in the training set, but some differences were not statistically significant in the validation set (Table 1). The RF scores in the training set showed good risk stratification; 418 cases (76.3%) were classified as very-low risk with no metastatic cases (Table 2). In addition, 6 LNM-positive cases (5 training and 1 validation cases) with minimal conventional histologic risk factors (only deep submucosal invasion, > 1 mm) showed moderate to high risk, with RF scores ranging from 0.814 to 0.900. On the other hand, in the validation set, 4 LNM-positive cases were classified as very-low risk, resulting in a 2.4% LNM-positive rate (Table 2). For endoscopically resected cases, all of the LNM-positive cases in the training set (*n* = 5) were classified as moderate- or high-risk, while those in validation set (*n* = 1) were classified as very low-risk (Supplementary Table 1). These results indicated that the model successfully predicted LNM, but some validation cases were difficult to predict.

**Table 1:** Comparison of important parameters for random forests.

Training set	LNM(−) (*n* = 505)	LNM( +) (*n* = 43)	*p*-value
Proportion of LNM (−) tiles (%)	50.18 ± 25.95	23.49 ± 15.32	< 0.00001*
Proportion of LNM (+) tiles (%)	49.82 ± 25.95	76.51 ± 15.32	< 0.00001*
AVE predictive value of LNM (−) tiles	0.644 ± 0.067	0.600 ± 0.025	< 0.00001*
AVE predictive value of LNM (+) tiles	0.640 ± 0.044	0.697 ± 0.052	< 0.00001*
Number of Group D tiles	32.67 ± 60.8	180.2 ± 253.6	< 0.001*
Number of Group E tiles	3.313 ± 9.738	32.68 ± 61.11	0.002*
Tumor location			
C,A,T	193	10	0.008**
D,S	138	9	
R	174	24	

Validation set	LNM(−) (*n* = 217)	LNM(+) (*n* = 18)	*p*-value
Proportion of LNM (−) tiles (%)	46.06 ± 25.08	35.52 ± 25.38	0.057*
Proportion of LNM (+) tiles (%)	53.94 ± 25.08	64.48 ± 25.38	0.057*
AVE predictive value of LNM (−) tiles	0.638 ± 0.064	0.616 ± 0.050	0.058*
AVE predictive value of LNM (+) tiles	0.644 ± 0.042	0.672 ± 0.056	0.031*
Number of Group D tiles	32.36 ± 52.28	116.2 ± 172.4	0.031*
Number of Group E tiles	3.152 ± 8.108	17.39 ± 29.87	0.033*
Tumor location			
C,A,T	69	4	1.00**
D,S	77	8	
R	71	6	

Average ± standard deviation. LNM(−), lymph node metastasis negative; LNM(+), lymph node metastasis positive; AVE, average, C, cecum; A, ascending colon; T, transverse colon; D, descending colon; S, sigmoid colon; R, rectum. *Student t-test, ** non-rectum versus rectum, Fisher exact test.

**Table 2:** Proportion of random forest (RF) scores.

RF Scores	LNM-negative	LNM-positive	LNM (%)
Training set (*n* = 548)			
0–0.7	418	0	0
0.7–0.8	43	5	10.4
0.8–0.9	39	29	42.6
0.9–	5	9	64.3
Validation set (*n* = 235)			
0–0.7	162	4	2.4
0.7–0.8	26	3	10.3
0.8–0.9	27	9	25.0
0.9–	2	2	50.0

LNM, lymph node metastasis.

### Whole-slide mapping of representative cases

**Figure 3:**
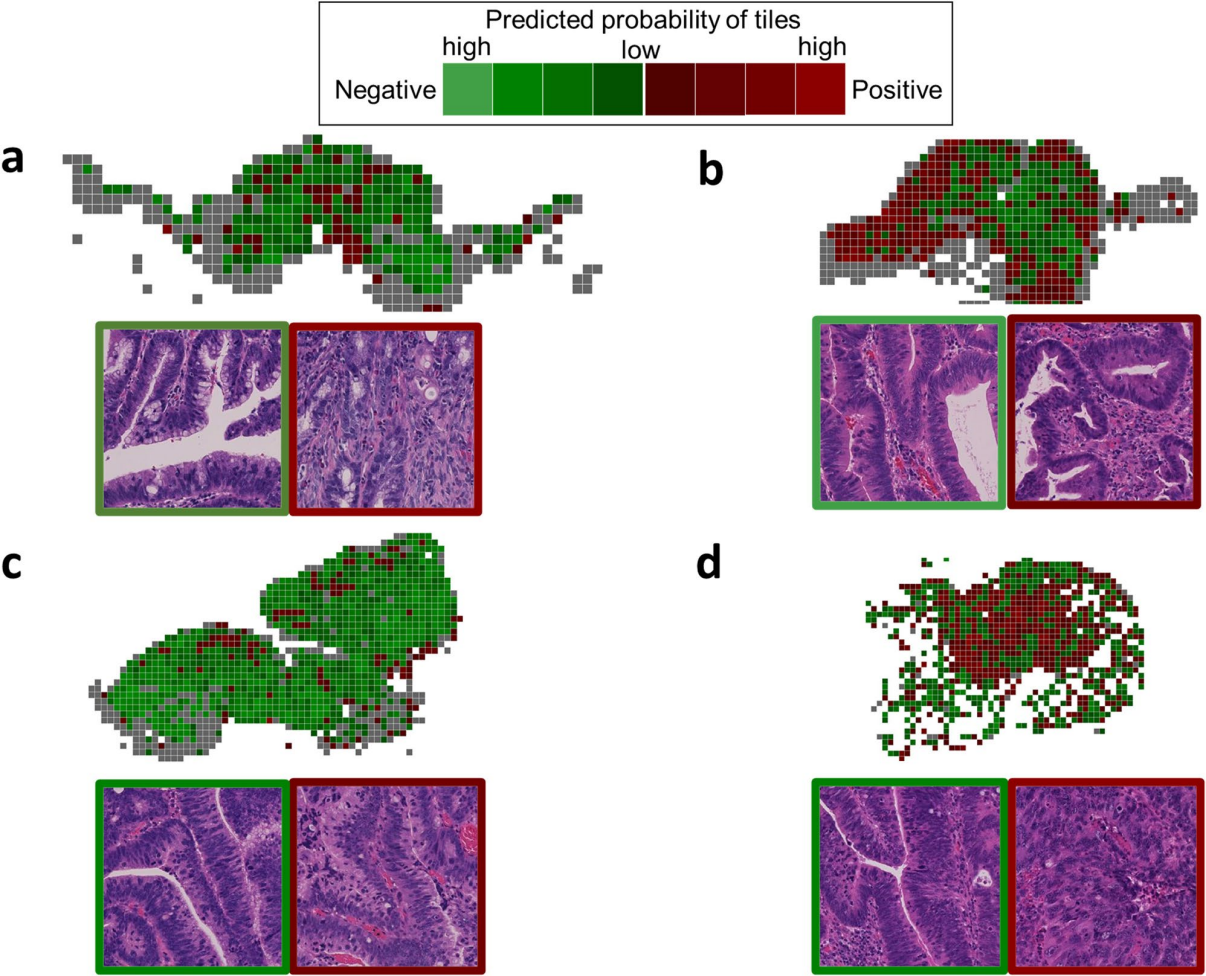
Four representative validation cases. Color mapping of tiles and representative tile images are shown. (**a**) LNM negative case with RF score of 0.0000, (**b**) LNM negative case with RF score of 0.9117, (**c**) LNM positive case with RF score of 0.0001, (**d**) LNM positive case with RF score of 0.9143. The red tiles correspond to positive predicted, and green tiles correspond to negative predicted. The color brightness represents the probability of each tile: the brighter the tile, the higher the probability. Gray tiles are classified as non-tumor by classifier #1. Scales: 1 tile equals to 273-µm square.

Cases with low RF scores had a small number of positive predicted tiles, while those with high RF scores had predominantly positive predicted tiles (Fig. 3). Cases that were LNM-negative but classified as high risk (i.e., false-positive) showed predominantly positive predicted tiles (Fig. 3b). In contrast, cases that were LNM-positive but classified as very-low risk (i.e., false-negative) tended to have many Group A and B tiles and few Group D and E tiles, resulting in low RF scores (Fig. 3c). Positive or negative predicted tiles tended to form clusters, but some single tiles existed within either cluster.

### Relationships between RF scores and conventional histologic risk factors

Cases were stratified by combining conventional histologic risk factors and their average RF scores are shown in Table 3. The lower the number of histologic risk factors, the lower the RF score. The important point is that the RF scores of LNM-positive cases with few conventional risk factors were consistently higher than those of LNM-negative cases, and some were statistically significant (Table 3). All of the LNM-positive cases harbor at least one risk factor, especially the deep submucosal invasion. There were 6 LNM-positive cases after endoscopic resection and all but one case showed moderate to high-risk (Supplementary Table 2A). On the other hand, LNM-positive cases with RF scores less than 0.8 harbor at least two histologic risk factors (Supplementary Table 2B). The average RF score was significantly higher in moderately and poorly differentiated tumors than in well differentiated tumors (Supplementary Table 3). When we compared cases with an RF score cutoff value of 0.7 and at least one histologic risk factor for conventional risk evaluation, the odds ratios for LNM were higher in both the training and validation sets than when using the conventional method (Supplementary Table 4A and 4B1). Though there were some false-negative cases in the machine learning model, the number of false-positive cases was low, resulting in better predictive odds. Similarly, the odds ratios of RF scores were higher than those when evaluating histologic-grade or poorly differentiated clusters. (Supplementary Table 4A, 4B2 and B3) Regarding conventional histologic-grade evaluation, interobserver disagreement was observed in some cancer images, while the machine learning model outputted correct predictive values for these cases (Supplementary Fig. 3).

**Table 3:** Correlation between conventional histologic risk factors and RF scores.

Histologic factors	Training set (*n* = 548)				p*	Validation set (*n* = 235)				p*
	LNM		Average RF Score			LNM		Average RF Score		
	(-)	( +)	LNM(-)	LNM( +)		(-)	( +)	LNM(-)	LNM( +)	
(None)	111	0	0.0921	NA	NA	44	0	0.25269	NA	NA
SM	172	7	0.2559	0.8597	< 0.001	65	1	0.23585	0.71252	NA
Ly	5	0	0.0960	NA	NA	2	0	0.00000	NA	NA
V	10	0	0.0855	NA	NA	4	0	0.17455	NA	NA
Por	0	0	NA	NA	NA	2	0	0.50594	NA	NA
BD	2	0	0.0000	NA	NA	0	0	NA	NA	NA
SM, Ly	17	1	0.2263	0.9371	NA	15	1	0.24825	0.53768	NA
SM, V	76	5	0.4162	0.8327	< 0.001	29	3	0.47607	0.59520	0.359
SM, Por	7	0	0.3492	NA	NA	1	0	0.89556	NA	NA
SM, BD	18	0	0.3193	NA	NA	18	0	0.32070	NA	NA
Ly, V	0	0	NA	NA	NA	2	0	0.48984	NA	NA
Ly, Por	1	0	0.0000	NA	NA	0	0	NA	NA	NA
Ly, BD	1	0	0.0000	NA	NA	0	0	NA	NA	NA
V, BD	0	1	NA	0.9317	NA	0	0	NA	NA	NA
Por, BD	1	0	0.0667	NA	NA	0	0	NA	NA	NA
SM, Ly, V	13	6	0.5098	0.8708	NA	2	1	0.57474	0.91431	NA
SM, Ly, Por	3	2	0.4094	0.8615	0.066	1	0	0.72192	NA	NA
SM, Ly, BD	14	2	0.3477	0.8778	< 0.001	5	2	0.19339	0.42831	0.335
SM, V, Por	2	0	0.0000	NA	NA	0	0	NA	NA	NA
SM, V, BD	17	2	0.4578	0.9114	< 0.001	9	1	0.51281	0.00010	NA
SM, Por, BD	2	1	0.3222	0.8200	NA	3	0	0.28134	NA	NA
Ly, Por, BD	0	1	NA	0.8839	NA	2	0	0.34964	NA	NA
V, Por, BD	1	0	0.7717	NA	NA	0	0	NA	NA	NA
SM, Ly, V, Por	3	0	0.6002	NA	NA	1	0	0.82216	NA	NA
SM, Ly, V, BD	10	6	0.4314	0.8566	0.004	4	2	0.70850	0.79865	0.121
SM, Ly, Por, BD	9	1	0.5763	0.8752	NA	5	1	0.19467	0.75696	NA
SM, V, Por, BD	5	1	0.2986	0.8440	NA	1	0	0.28337	NA	NA
SM, Ly, V, Por, BD	5	7	0.5555	0.8759	0.052	3	5	0.50640	0.69660	0.274

LNM, lymph node metastasis; RF, random forest; SM, deep submucosal invasion; Ly, lymphatic invasion; V, venous invasion; Por, poorly differentiated clusters; BD, high-grade tumor budding; NA, not applicable. *Student t-test.

## Discussion

We present an LNM prediction model for T1 CRC with explanatory histologic images for visualization of the basis of the predictive values. We combined CNN and RF to visualize the learning and deciding algorithms for predicting LNM, providing persuasive logic for pathologists and clinicians.

Prognostic prediction of CRC by artificial intelligence has been attempted using several approaches. Some researchers developed a machine-learning algorithm based on the conventional clinicopathologic information, including histologic factors of CRC^27–29^. These studies were performed using several machine-learning algorithms. A possible advantage of utilizing conventional clinicopathologic information is the readability of the analysis results, which clinicians can use to explain to patients why the machine returned certain results. This point is critical for the application of machine-learning techniques in the field of clinical medicine because understandable evidence is the most important factor for both patients and clinicians in making a clinical decision. On the other hand, diagnostic reproducibility is also critical for a stable prediction model. In particular, interobserver disagreement of histopathologic risk factors for predicting LNM of T1 CRC has remained problematic and may not be completely resolved. Because artificial intelligence can manage a huge amount of information, direct analysis of histologic images may contribute to developing a stable prediction model. Interestingly, our LNM prediction model mimicked histologic-grade evaluation for tile images. Since the interobserver disagreement for histologic grades is difficult to solve as described in previous reports^30–32^, and we also demonstrated in Supplementary Fig. 3, our model can provide more stable and reliable prediction results by skipping this evaluation step.

CNN is an ideal method for classifying histologic images into various categories that has been used for classifying histologic findings in many previous studies to predict gene alterations and patient prognosis^18,21,23,33^. Supervised machine-learning with appropriately labeled data enables complex histologic analyses by extracting features of colored images. Skrede et al.^25^ revealed that a combination of 2 neural networks for tile images can predict the prognosis of CRC through analyses of a large multicenter cohort. Most histologic analyses with CNN have been performed for small tile images with hundreds of pixels squares. Though it is difficult to understand the thorough process of CNN for returning its values, a visualizing method such as Grad-CAM may help to understand the histologic machine-learning theory^33,34^. This method may clarify the importance of the pixels within a tile image. Because most carcinomas, however, including CRC, show intra-tumoral histologic heterogeneity^35^, it is not enough to understand whole tumor characteristics by analyzing each tile. Learning how to extract features from a set of tile images is important to give clinicians and patients valuable information, such as a cancer prognosis.

RF has been used for predicting the prognosis of various cancers on the basis of radiologic and histopathologic images^15,17,20^. The classification ability of the RF score depends on the given parameters, such as number of tile images and predictive probabilities, and differs from that of classification by CNN, which requires only input images. RF can output the importance of parameters of any type, which plays critical role for converting characteristics of various tile images into a simple score through the understandable logic of decision trees. Histologic WSIs contain a lot of information, and most solid tumors exhibit histologic heterogeneity; thus, visualization of the decision process for predicting prognosis may provide a novel approach for histologic classification. In this study, CNN calculated and extracted characteristic images affecting the risk of metastasis (Group D and E), which contained poorly differentiated cancer clusters and desmoplastic stroma. Furthermore, RF indicates the proportion of LNM-positive or LNM-negative tiles, and their average probabilities were important for predicting LNM for cases. RF scores of our model tended to be higher in the LNM positive cases, indicating that this method may produce different results from the conventional method. Moreover, RF can calculate variables other than image features, such as tumor location, which provides researchers some additional options to improve the accuracy of the model. Tumor location is a risk stratification factor for T1 CRC^13,36,37^. Rectal cancers are more likely to metastasize than colon cancers^36^, and our results revealed similar tendency (Table 1). According to this fact, in our prediction model, RF indicated the importance of the tumor location, but no local predilection was demonstrated in the validation set (Table 1). This phenomenon may be attributed to the small number of LNM-positive cases in the validation set, having a negative effect on the LNM prediction. It is important to note that the large amount of outcome data for predicting LNM cases with RF was much smaller than the amount of data from millions of tile images, which require oversampling the data of LNM-positive cases to fill the tenfold gap against those of LNM-negative cases and avoid inappropriate predilection to the majority group. In contrast, the cancer tile images for the LNM-positive group numbered nearly 50 thousand, which seemed to be enough to avoid overfitting in CNN without over- or under-sampling, as indicated by the learning curves (Supplementary Fig. 1 and 2).

Because most T1 CRC patients are curable by surgical treatment even if overtreated, it could be worse if the tumor relapsed locally or by distant metastases^7^. In this context, false-negative cases should be minimized to avoid life-threatening relapse. In this study, we successfully developed a prediction model extracting 76.3% of truly negative case with low RF scores in the training set. On the other hand, 4 LNM-positive cases in the validation set were predicted to be very-low risk. All 4 of these cases contained many negative-prediction tiles; therefore, the most probable cause of the false-negative would be a lack of training tile images of LNM-positive cases with the features of these cases. To solve this matter, additional images of LNM-positive T1 CRC should be provided. The variability of input data among different institutions is also important. The quality of histological images depends on several factors such as the resection procedure, preparation of paraffin sections, staining procedure, and digitizing steps. In this study, we analyzed assembled tile images originating from H&E stained whole-slide images. The image quality or color balance may differ from that in other institutions, even if the staining procedures and digitizing steps are fully automated. These factors may decrease the performance of the prediction model and should be carefully evaluated before applying this method in clinical practice. Because the factors are quite complex and some of their variabilities are difficult to resolve, a multicenter validation study should be performed to clarify the differences of the factors, and to understand how to maintain the precision of the model in any situation. These are limitations of this single institutional study, and further investigation should be performed in a large-scale multicentral cohort study. Another limitation of this study is that the followed up patients with no LNM by periodic colonoscopy and/or abdominal computed tomography scanning for 5 years after initial resection may be misdiagnosed relapsing LNM, will result in false-negative or false-positive that the ground truth data can be changed in such cases.

In conclusion, we introduced a machine-learning model for predicting LNM of T1 CRC, which enabled risk-stratification of cases without any conventional histologic risk assessment. Our method will be a useful tool for clinicians and patients to understand the decision process of artificial intelligence through visualizing characteristic histologic images.

## Methods

### Case recruitment

A total of 855 consecutive T1 CRC resected either endoscopically or surgically at The Cancer Institute Hospital between 2005 and 2015, were initially recruited in this study. Of these 855 cases, 68 were excluded because of inadequate sample conditions (e.g., non-enbloc resections), and 787 cases underwent further analyses. Among the patients, 61 cases (7.8%) showed metastasis in dissected lymph nodes (LNM-positive). Among the patients treated with endoscopic resection, 119 patients (43.1%) underwent additional intestinal resection with lymph node dissection, and 157 (56.9%) patients were followed up for 5 years by periodic colonoscopy and/or abdominal computed tomography scanning, based on the guidelines of Japanese Society for Cancer of the Colon and Rectum^6^. The cases were randomly sorted by computer to divide the cases into a training set (*n* = 551) and test set (*n* = 236). The LNM rate was equivalent for both datasets. Patient characteristics is described in Supplementary Table 5. To develop the histologic-type classifier, we randomly recruited 22 T1 CRC cases and an additional 100 T2/3 CRC cases that were surgically resected in 2008 and 2009.

### Histologic evaluation and digitizing

Serial 2-mm- to 5-mm-thick tissue sections of the whole lesion were cut from resected specimens fixed with 20% buffered formalin and embedded in paraffin; 3-μm-thick sections were then prepared for staining. Each section was stained with H&E. For each case, all cancer-containing sections showing submucosal invasion were included. Conventional histologic evaluation of the depth of submucosal invasion (≥ 1000 µm), poorly differentiated or mucinous carcinoma, lymphatic invasion, venous invasion, and tumor budding was performed by 2 pathologists (M.T, H.K), to disclose the clinicopathologic characteristics of the cases (Supplementary Table 5), basically according to the Japanese Society for Cancer of the Colon and Rectum criteria^6^. In addition, we also evaluated histologic grades considering predominant tumor component, as follows: well, moderately and poorly differentiated. We also performed histologic grading by 3 pathologists (M.T, N.Y, H.K) for representative cases to demonstrate interobserver disagreement. Deep submucosal invasion was defined as tumor invasion of the submucosal layer with a depth of at least 1000 µm. The presence of poorly differentiated clusters was evaluated according to the criteria of Ueno et al.^38^.

**Figure 4:**
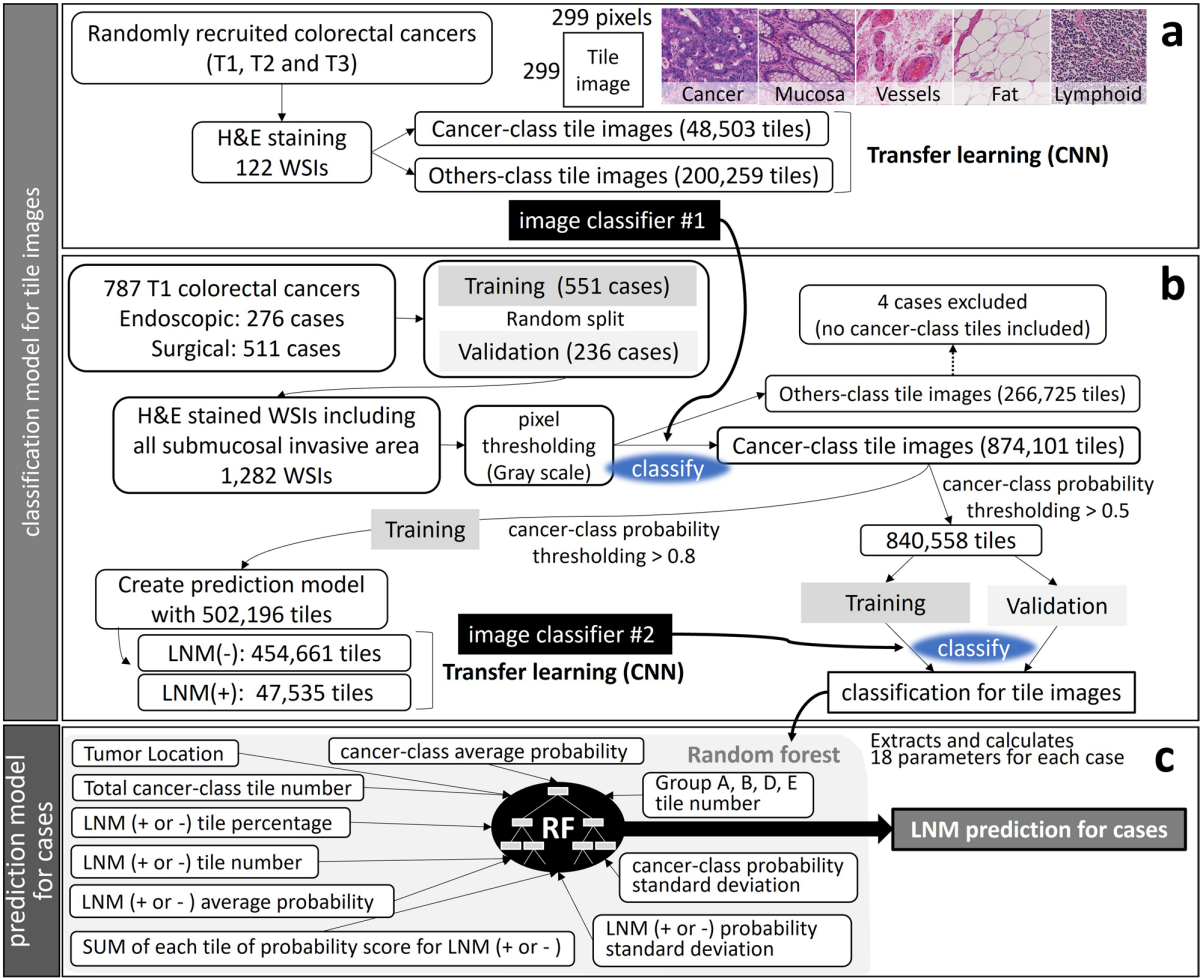
Study workflow for developing lymph node metastasis prediction model for tile images and for the cases using 2 convolutional neural networks (CNN) and a random forest (RF) algorithm. (**a**) Creating image classifier #1 by a neural network with cancer or other tissue labeled tile images. (**b**) Creating image classifier #2 by re-training the neural network with lymph node metastasis positive or negative labeling based on patient outcome. LNM(−), negative for lymph node metastasis; LNM(+), positive for lymph node metastasis; SUM, summary. (**c**) Random forest classifier determines the predictive values for lymph node metastasis based on several parameters of the tile images.

Whole-slide images (WSIs) of the sections were obtained by a digital slide scanner (NanoZoomer, Hamamatsu Photonics, Hamamatsu, Japan). The WSIs were cut into non-overlapping small tile images of 299-pixel squares (equal to a 273-µm square). To ensure that the image contained at least one nucleated cell, each tile image was temporarily converted to gray-scale (0–255), and the image containing the minimum pixel value of 110 was adopted for further image analysis (pixel thresholding, Fig. 4b).

### Statistical analysis

The primary endpoint of this study was LNM, and 5-year disease-free survival was defined as no metastases for cases without surgical treatment. Receiver operating characteristics (ROC) curves were used to estimate the predictive power of the model. The balanced error rate of the ROC curve was considered an optimal cut-off value for determining the accuracy of the model. The area under the ROC curve (AUC) was defined as the predictive ability of the models. Qualitative factors were analyzed with the Fisher exact test and odds ratios were calculated. Quantitative factors were analyzed with the Student t-test. A *P*-value of less than 0.05 was considered statistically significant in both analyses. We conducted all analyses using R version 3.6.2^39^.

### Neural network machine-learning models

First, we created a histologic-type classifier by CNN to enable the efficient selection of cancer tiles. The tile images from 122 WSIs of randomly selected CRCs were labeled with “cancer” or 9 other labels, including non-tumoral mucosa, hyperplastic mucosa, adenoma, lymphoid tissue, smooth muscle, vessels, fat, nerve, and non-material background. The number of cancer-labeled images was 48,503, and that of the others was 200,259. A deep-learning pre-trained model, MobileNet V2^40^, was re-trained to recognize 10 different tissue types to develop image classifier #1 (Fig. 4a). Labeling of tile images originating from WSIs was performed by 1 pathologist (M.T), using OpenSlide library running on Python 3.7^41^.

Second, the tile images from 1282 WSIs of T1 CRC underwent pixel thresholding and were classified with image classifier #1. Each image was re-labeled by the classifier with the probable histologic type and probability for classifying. Four cases were excluded because no cancer-class tiles were detected, therefore 548 training cases and 235 validation cases were applied to further analyses. Among the cancer-class images, we adopted images with a probability > 0.8 to create the LNM prediction model. These tile images were re-labeled again with the primary endpoint and re-trained to develop image classifier #2, which classified whether or not a cancer tile image was likely to metastasize.

Then, cancer-class tile images of both the training set and validation set with a probability > 0.5 (840 558 tiles) were classified by image classifier #2 and re-labeled with the probable LNM status and probability for metastasis. We defined the tile images according to the probability as follows: those with non-metastatic probability > 0.9 as Group A, those with non-metastatic probability between 0.8 and 0.9 as Group B, those with metastatic probability between 0.8 and 0.9 as Group D, those with metastatic probability > 0.9 as Group E, and those with either probability < 0.8 as Group C. We also defined probability score summary of CNN, which can be obtained by multiplying the cancer-class probability with the metastatic probability for each tile, and summarized for case. We tuned the classifiers by learning iteration and learning rate to obtain maximum accuracy and minimum loss while re-training. The loss was evaluated by cross entropy. The workflow from WSIs to complete classifying tile images is shown in Fig. 4b.

### Random-forest machine learning model

To establish the LNM prediction model for the cases, we loaded data from the tile images of the training set onto a machine-learning tool, the Scikit-learn on Python 3.6. We selected a random forest (RF) classifier machine-learning algorithm to minimize the effect of overfitting. Because the number of LNM-positive (*n* = 43) and LNM-negative (*n* = 505) cases in the training dataset had a tenfold gap, we randomly oversampled LNM-positive cases to equalize the number of cases applied to the RF model. The data contained 18 parameters as follows: tumor location; total number of cancer-class tiles; number of tiles classified as metastatic or non-metastatic; number of Group A, B, D, E tiles; percentages of tiles classified as metastatic or non-metastatic; average probabilities; standard deviations of cancer-class probabilities and metastatic or non-metastatic probabilities; and probability score summary for each tile (Fig. 4c). Tumor locations were grouped into 3 parts as follows; (1) cecum, ascending and transverse colon, (2) descending and sigmoid colon, and (3) rectum. The RF algorithm used was based on a previous report^42,43^. In brief, the RF randomly collected these parameters for creating dozens of decision trees. Teacher data of the primary endpoint were given to the RF, and the importance of parameters that minimize the impurity for separating LNM-positive or LNM-negative cases was calculated. The importance of each parameter was averaged in all the trees, and finally, the output predictive value of LNM for the case was determined. To reduce false-negative metastatic cases, we established 500 RFs to obtain a higher AUC for each RF and adopted the top 20 RFs for case analyses. The importance of the parameters of these RFs was averaged to show how the algorithm determined the score. The RFs returned values of metastatic probability between 0 and 1, and the maximum value of each case was adopted as the RF score. We tuned the RF to maximize the AUC for predicting LNM in all cases by adjusting the number and depth of the trees. We defined the RF scores as follows: 0–0.7, very-low risk; 0.7–0.8, low risk; 0.8–0.9, moderate risk; and 0.9–1.0, high risk.

### Ethics

The experimental protocols in this study were approved by the Ethics Committee of the Cancer Institute, Japanese Foundation for Cancer Research (approved number: 2020-1045). The written informed consent was obtained from all participants. This study was performed in accordance with the Declaration of Helsinki.

## Supplementary Information

Supplementary Figure 1.

Supplementary Figure 2.

Supplementary Figure 3.

Supplementary Table 1.

Supplementary Table 2.

Supplementary Table 3.

Supplementary Table 4.

Supplementary Table 5.
