# Supplementary figures and images for: Prediction of lymph node metastasis in early colorectal cancer based on histologic images by artificial intelligence
Source: Sci Rep. 2022 Feb 22;12:2963. doi: 10.1038/s41598-022-07038-1 (PMC8863850; doi:10.1038/s41598-022-07038-1)

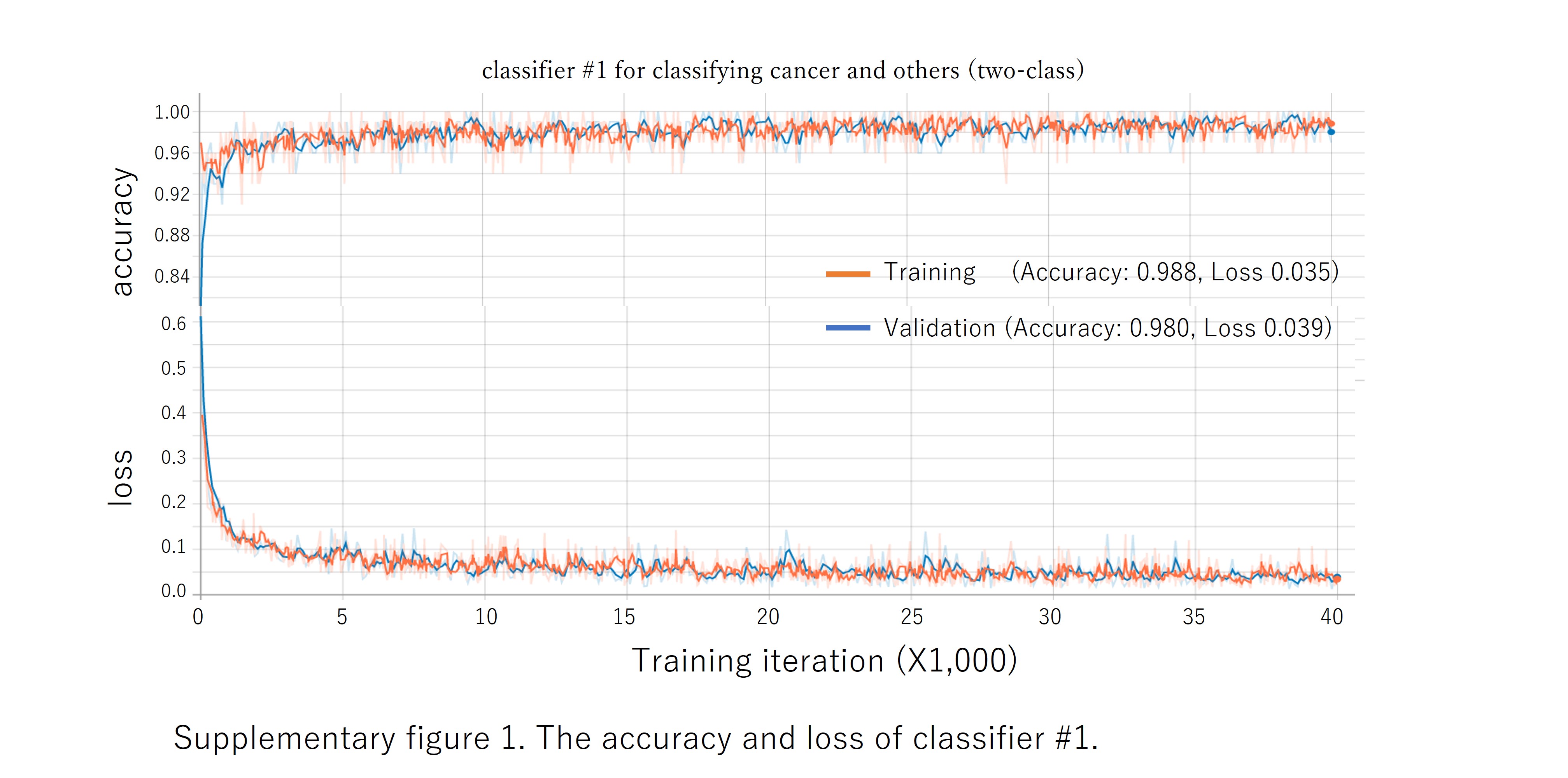

Supplement: Supplementary file 1 — Supplementary Figure 1. [file 41598_2022_7038_MOESM1_ESM.jpg]

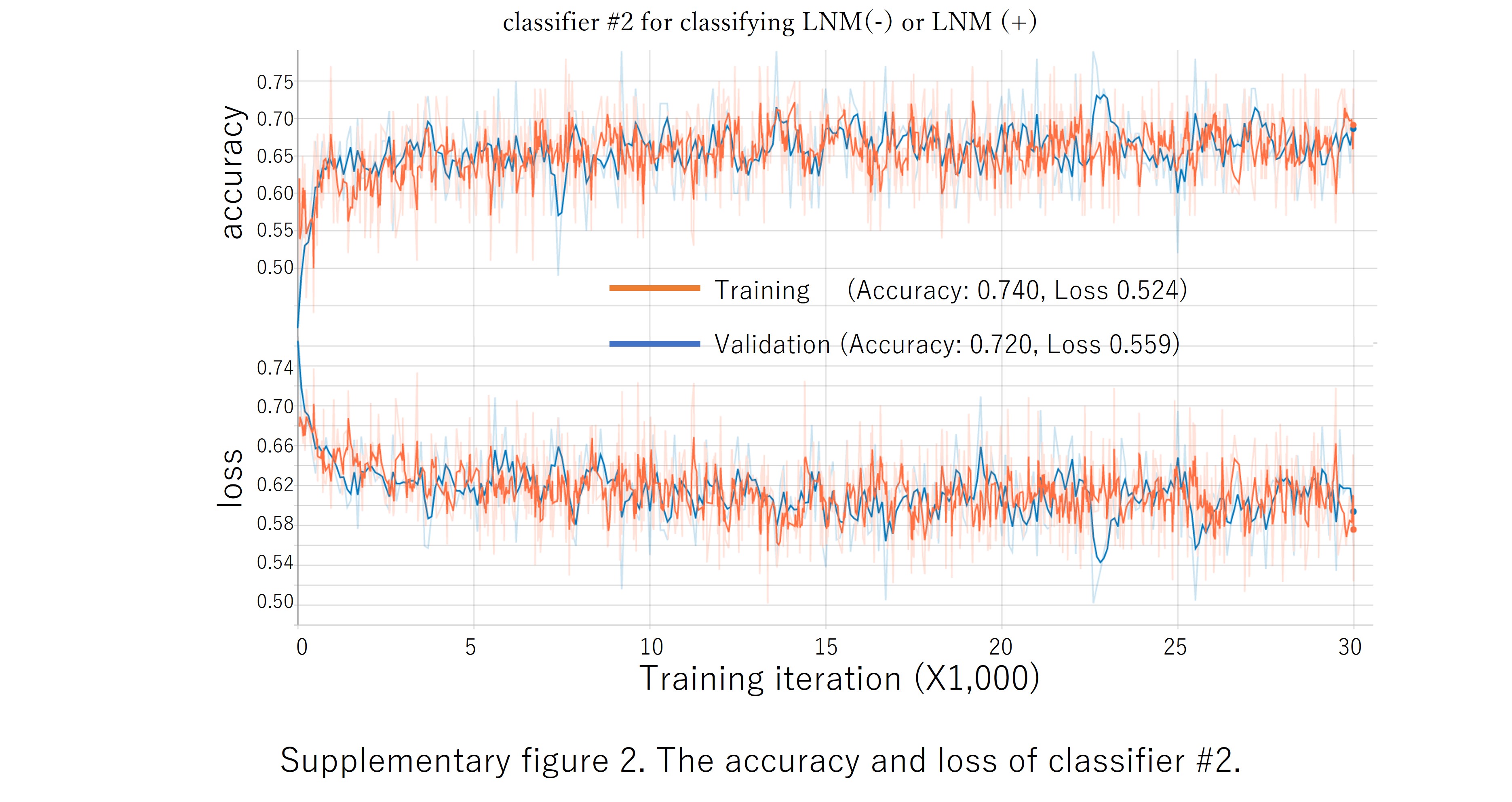

Supplement: Supplementary file 2 — Supplementary Figure 2. [file 41598_2022_7038_MOESM2_ESM.jpg]

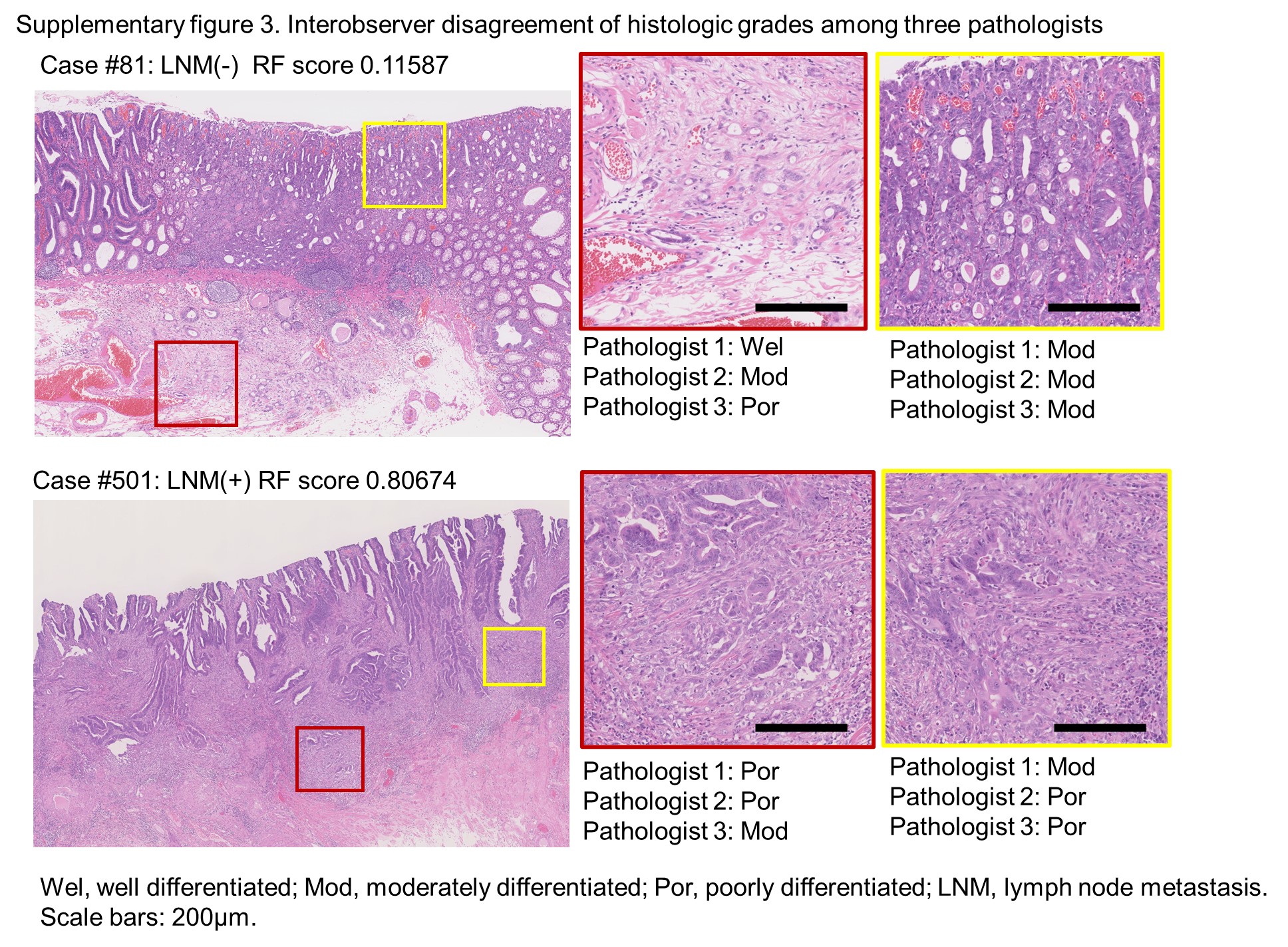

Supplement: Supplementary file 3 — Supplementary Figure 3. [file 41598_2022_7038_MOESM3_ESM.jpg]
