# Supplementary material for: Prediction of lymph node metastasis in early colorectal cancer based on histologic images by artificial intelligence
Source: Sci Rep. 2022 Feb 22;12:2963. doi: 10.1038/s41598-022-07038-1 (PMC8863850; doi:10.1038/s41598-022-07038-1)
Supplement: Supplementary file 4 — Supplementary Table 1. [file 41598_2022_7038_MOESM4_ESM.docx]

Supplementary Table 1. Proportion of random forest (RF) scores for cases with initially endoscopic resection

| RF Scores | LNM-negative | LNM-positive | LNM (%) |
| --- | --- | --- | --- |
| Training set (n=194) | | | |
| 0-0.7 | 181 | 0 | 0 |
| 0.7-0.8 | 5 | 0 | 0 |
| 0.8-0.9 | 3 | 4 | 57.1 |
| 0.9- | 0 | 1 | 100 |
| Validation set (n=77) | | | |
| 0-0.7 | 71 | 1 | 1.4 |
| 0.7-0.8 | 5 | 0 | 0.0 |
| 0.8-0.9 | 0 | 0 | NA |
| 0.9- | 1 | 0 | 0.0 |

LNM, lymph node metastasis; RF, random forest; NA, not applicable.
