# Supplementary material for: Prediction of lymph node metastasis in early colorectal cancer based on histologic images by artificial intelligence
Source: Sci Rep. 2022 Feb 22;12:2963. doi: 10.1038/s41598-022-07038-1 (PMC8863850; doi:10.1038/s41598-022-07038-1)
Supplement: Supplementary file 5 — Supplementary Table 2. [file 41598_2022_7038_MOESM5_ESM.docx]

Supplementary Table 2. RF score and conventional histologic risk factors of LNM-positive cases

| Case ID | training/ validation | RF score | SM | Ly | V | Por | BD |
| --- | --- | --- | --- | --- | --- | --- | --- |
| A. Endoscopic resection with additional surgery | | | | | | | |
| #479 | validation | 0.017 | + | + | - | - | + |
| #572 | training | 0.825 | + | - | - | - | - |
| #53 | training | 0.867 | + | + | + | - | - |
| #73 | training | 0.881 | + | + | + | - | - |
| #289 | training | 0.885 | + | - | - | - | - |
| #19 | training | 0.917 | + | + | + | - | - |
|  | | | | | | | |
| B. RF score less than 0.8 (very-low or low risk) | | | | | | | |
| #635 | validation | 0.001 | + | - | + | - | + |
| #479 | validation | 0.017 | + | + | - | - | + |
| #673 | validation | 0.028 | + | - | + | - | - |
| #32 | validation | 0.131 | + | + | + | + | + |
| #353 | validation | 0.704 | + | + | - | - | - |
| #242 | validation | 0.757 | + | + | - | + | + |
| #557 | validation | 0.774 | + | + | + | - | + |
| #419 | training | 0.773 | + | + | + | + | + |
| #596 | training | 0.781 | + | - | + | - | - |
| #619 | training | 0.785 | + | + | + | - | + |
| #412 | training | 0.788 | + | + | + | - | - |
| #95 | training | 0.796 | + | - | + | - | - |

LNM, lymph node metastasis; RF, random forest; SM, deep submucosal invasion; Ly, lymphatic invasion; V, venous invasion; Por, poorly differentiated clusters; BD, high-grade tumor budding; NA, not applicable.
