# Supplementary material for: Prediction of lymph node metastasis in early colorectal cancer based on histologic images by artificial intelligence
Source: Sci Rep. 2022 Feb 22;12:2963. doi: 10.1038/s41598-022-07038-1 (PMC8863850; doi:10.1038/s41598-022-07038-1)
Supplement: Supplementary file 6 — Supplementary Table 3. [file 41598_2022_7038_MOESM6_ESM.docx]

Supplementary Table 3. Relationship between RF scores and histologic grades

|  | RF score  (average±standard deviation) | p |
| --- | --- | --- |
| Training set (n=548) | | |
| Well differentiated | 0.26±0.32 | <0.0001* |
| Moderately differentiated | 0.43±0.33 |  |
| Poorly differentiated | 0.50±0.39 |  |
| Validation set (n=235) | | |
| Well differentiated | 0.29±0.32 | <0.0001* |
| Moderately differentiated | 0.53±0.34 |  |
| Poorly differentiated | 0.51±0.33 |  |

*Student t-test. Compared well differentiated to moderately and poorly differentiated.
