# Supplementary material for: Prediction of lymph node metastasis in early colorectal cancer based on histologic images by artificial intelligence
Source: Sci Rep. 2022 Feb 22;12:2963. doi: 10.1038/s41598-022-07038-1 (PMC8863850; doi:10.1038/s41598-022-07038-1)
Supplement: Supplementary file 7 — Supplementary Table 4. [file 41598_2022_7038_MOESM7_ESM.docx]

Supplementary Table 4. Comparison of artificial intelligence model and conventional histologic evaluation

A. Artificial intelligence

|  | LNM-negative | LNM-positive | Odds ratios  (95% CI) | p |
| --- | --- | --- | --- | --- |
| Training set (n=548) | | | | |
| Very low risk  (RF Scores 0-0.7) | 418 | 0 | 416.1  (25.4-6823) | <0.0001 |
| At least low risk  (RF Scores 0.7-1.0) | 87 | 43 |  |  |
| Validation set (n=235) | | |  |  |
| Very low risk  (RF Scores 0-0.7) | 162 | 4 | 10.3  (3.3-32.6) | <0.0001 |
| At least low risk  (RF Scores 0.7-1.0) | 55 | 14 |  |  |

B. Conventional histologic risk evaluation

1. Five risk factors (deep submucosal invasion, lymphatic invasion, venous invasion, poorly differentiated clusters and high-grade tumor budding)

|  | LNM-negative | LNM-positive | Odds ratios | p |
| --- | --- | --- | --- | --- |
| Training set (n=548) | | | | |
| No histologic risk factors | 111 | 0 | 24.6  (1.5-402.6) | 0.0001 |
| At least one risk factor | 394 | 43 |  |  |
| Validation set (n=235) | | | | |
| No histologic risk factors | 44 | 0 | 9.28  (0.6-157.0) | 0.0504 |
| At least one risk factor | 173 | 18 |  |  |

2. Histologic grade

|  | LNM-negative | LNM-positive | Odds ratios | p |
| --- | --- | --- | --- | --- |
| Training set (n=548) | | | | |
| Well differentiated | 356 | 21 | 2.50*  (1.34-4.69) | 0.0055* |
| Moderately differentiated | 146 | 19 |  |  |
| Poorly differentiated | 3 | 3 |  |  |
| Validation set (n=235) | | | | |
| Well differentiated | 151 | 10 | 1.83*  (0.69-4.85) | 0.2898* |
| Moderately differentiated | 63 | 8 |  |  |
| Poorly differentiated | 3 | 0 |  |  |

* Compared well differentiated to moderately and poorly differentiated.

3. Poorly differentiated clusters only

|  | LNM-negative | LNM-positive | Odds ratios | p |
| --- | --- | --- | --- | --- |
| Training set (n=548) | | | | |
| PDC (-) | 466 | 30 | 5.18  (2.50-10.7) | <0.0001 |
| PDC (+) | 39 | 13 |  |  |
| Validation set (n=235) | | | | |
| PDC (-) | 199 | 11 | 7.04  (2.43-20.4) | 0.001 |
| PDC (+) | 18 | 7 |  |  |

PDC, poorly differentiated clusters
