# Supplementary material for: Prediction of lymph node metastasis in early colorectal cancer based on histologic images by artificial intelligence
Source: Sci Rep. 2022 Feb 22;12:2963. doi: 10.1038/s41598-022-07038-1 (PMC8863850; doi:10.1038/s41598-022-07038-1)
Supplement: Supplementary file 8 — Supplementary Table 5. [file 41598_2022_7038_MOESM8_ESM.docx]

Supplementary Table 5. Patient characteristics

|  |  | LNM(-) |  | LNM(+) |  | p-value |  |
| --- | --- | --- | --- | --- | --- | --- | --- |
| Sex | Female | 314 |  | 32 |  | 0.1820 | * |
|  | Male | 408 |  | 29 |  |  |  |
|  |  |  |  |  |  |  |  |
| Initial treatment | Endoscopy | 265 |  | 6 |  | <0.0001 | * |
|  | Surgery | 457 |  | 55 |  |  |  |
|  |  |  |  |  |  |  |  |
| Age | Average | 64.84 |  | 62.79 |  | 0.0916 | ** |
|  | STD | 11.89 |  | 11.35 |  |  |  |
|  |  |  |  |  |  |  |  |
| Location | C, A, T | 262 |  | 14 |  | 0.0246 | * |
|  | D, S | 215 |  | 17 |  |  |  |
|  | R | 245 |  | 30 |  |  |  |
|  |  |  |  |  |  |  |  |
| Tumor size (mm) | Average | 20.84 |  | 22.28 |  | 0.1763 | ** |
|  | STD | 12.77 |  | 11.33 |  |  |  |
|  |  |  |  |  |  |  |  |
| Histologic type | TUB | 713 |  | 58 |  | 0.0272 | * |
| (predominant) | POR | 6 |  | 3 |  |  |  |
|  |  |  |  |  |  |  |  |
| Invasion depth (µm) | Average | 2380.8 |  | 3528.8 |  | <0.0001 | ** |
|  | STD | 1739.5 |  | 1643.7 |  |  |  |
|  |  |  |  |  |  |  |  |
| Lymphatic invasion | negative | 599 |  | 22 |  | <0.0001 | * |
|  | positive | 108 |  | 28 |  |  |  |
|  |  |  |  |  |  |  |  |
| Vascular invasion | negative | 525 |  | 20 |  | <0.0001 | * |
|  | positive | 175 |  | 35 |  |  |  |
|  |  |  |  |  |  |  |  |
| Poorly differentiated | negative | 665 |  | 41 |  | <0.0001 | * |
| clusters | positive | 57 |  | 20 |  |  |  |
|  |  |  |  |  |  |  |  |
| Tumor budding | Low-grade | 586 |  | 28 |  | <0.0001 | * |
|  | High-grade | 135 |  | 33 |  |  |  |

STD, standard deviation; C, cecum; A, ascending colon; T, transverse colon; D, descending colon; S, sigmoid colon; R, rectum; TUB, tubular adenocarcinoma; POR, poorly differentiated adenocarcinoma; LNM(-), negative for lymph node metastasis; LNM(+), positive for lymph node metastasis. *Fisher exact test, **Student t-test.
